# Gammaherpesvirus RNAs Come Full Circle

**DOI:** 10.1128/mBio.00071-19

**Published:** 2019-04-02

**Authors:** Nathan A. Ungerleider, Scott A. Tibbetts, Rolf Renne, Erik K. Flemington

**Affiliations:** a Department of Pathology, Tulane University School of Medicine, Tulane Cancer Center, New Orleans, Louisiana, USA; b Department of Molecular Genetics and Microbiology, University of Florida, Gainesville, Florida, USA; University of Wisconsin–Madison; University of Texas Health Science Center at Houston

**Keywords:** EBV, KSHV, MHV68, circRNA, circular RNA, gammaherpesvirus, lymphocryptovirus, rLCV, rhadinovirus

## Abstract

After an adaptive immune response is mounted, gammaherpesviruses achieve persistence through the utilization of viral noncoding RNAs to craft a suitable host cell environment in an immunologically transparent manner. While gammaherpesvirus long noncoding RNAs (lncRNAs) and microRNAs have been recognized for some time and have been actively investigated, a recent spate of reports have now identified repertoires of the circular RNA (circRNA) class of noncoding RNAs in both the lymphocryptovirus and rhadinovirus genera of gammaherpesviruses. Despite the recent nature of these findings, the detection of circRNAs across viruses and viral gene expression programs, the conservation of some viral circRNAs, and their detection in the clinical setting already raises the spectrum of functional importance in gammaherpesvirus biology and associated malignancies. Here, we provide an overview of currently known gammaherpesvirus circular RNAs and discuss reported physical and contextual properties that may be germane to future functional studies. With the Epstein-Barr virus (EBV) circRNAome being the most extensively studied to date, our discussions will be weighted toward EBV circRNAs while also addressing circRNAs discovered in the rhesus macaque lymphocryptovirus (rLCV), the Kaposi’s sarcoma herpesvirus (KSHV), and the murid gammaherpesvirus 68 (MHV68). We hope that this will help set the stage for future investigations into the functions and relevance of this new class of viral noncoding RNAs in infection and disease.

## Abstract (precis)

After an adaptive immune response is mounted, gammaherpesviruses achieve persistence through the utilization of viral noncoding RNAs to craft a suitable host cell environment in an immunologically transparent manner. While gammaherpesvirus long noncoding RNAs (lncRNAs) and microRNAs have been recognized for some time and have been actively investigated, a recent spate of reports have now identified repertoires of the circular RNA (circRNA) class of noncoding RNAs in both the lymphocryptovirus and rhadinovirus genera of gammaherpesviruses.

## INTRODUCTION

After initial infection, gammaherpesviruses persist in the host and evade immune clearance in part through minimizing viral gene expression. Nevertheless, their tenure in the host during these immune evasion phases is not idle. Rather, they continue to remodel their host environment through viral noncoding RNA interactions with cell signaling pathways to facilitate host cell alterations without eliciting immune recognition. This is accomplished through the use of both long noncoding RNAs (lncRNAs) and small noncoding RNAs, with every gammaherpesvirus analyzed to date bearing a broad repertoire of the microRNA (miRNA) class of small noncoding RNAs (reviewed in references 1 and 2).

The extent of gammaherpesvirus transcriptome complexity that is packed into these relatively small genomes has only recently become appreciated despite decades of previous study (1–9). Although this work has uncovered diverse transcript types, the discovery of novel viral noncoding RNAs is particularly important for our understanding of how gammaherpesviruses interact with their host environment during the latent phases of infection. Circular RNAs (circRNAs) (10–12) are a conserved class of predominantly noncoding RNAs that have gained increased attention in recent years (13–16). circRNAs are produced by backsplicing of a 3′ splice donor to an upstream 5′ splice acceptor, generating a closed circRNA molecule through a 3′–5′ covalent linkage. While circRNAs were discovered many years ago as interesting anomalies (10–12), recent high-throughput sequencing approaches have facilitated their identification across all kingdoms of life (13–17). With circRNAs functioning primarily as noncoding RNAs (18), the recent discovery of circRNAs in the gammaherpesviruses, Epstein-Barr virus (EBV), rhesus lymphocryptovirus (rLCV), Kaposi’s sarcoma herpesvirus (KSHV), and murid herpesvirus 68 (MHV68) is not surprising (19–23). However, this class of noncoding RNAs has gone unappreciated for many years, and their recent discovery opens our eyes to a whole new set of viral cell signaling effectors that must be investigated to understand the biology of these viruses and their contributions to gammaherpesvirus-associated cancers.

## CURIOUS SPLICING MISTAKES OR FUNCTIONAL SIGNALING EFFECTORS?

More than a thousand circRNA papers have been published in the last 5 years, with the vast majority of these addressing discovery, tissue type, and/or pathology correlations, diagnostic potential, or methodological issues rather than function. Important insights into the functions of circRNAs have, in fact, been made over the past few years (reviewed in reference 18), but there are unique challenges to studying the function of circRNAs that have impeded progress. One challenge is that circRNAs contain a molecular makeup nearly identical to that of their linear coexpressed counterparts, with the only unique physical feature of the circRNAs being the juxtaposed ends at the backsplice junction. Therefore, specific detection or knockdown approaches must utilize methods that leverage this single unique feature of circRNAs. Further, ectopic expression is often not appropriate to study the function of circRNAs, because (i) the functions of circRNAs are dictated in part by the loading of splicing factor cargo from their natural introns (24, 25), and (ii) some circRNAs function in *cis* on their gene of origin (26). Thus, not surprisingly, being the early stages of gammaherpesvirus circRNA investigation, there is only scant insight into their functions. At this point, we can consider a question that still lingers in the minds of many scientists pondering the significance of cellular circRNAs: do most of these backsplice events reflect curious splicing mistakes that serve no function, or are viral circRNAs evolutionarily conserved signaling effectors? While this is a valid question, there are already hints that many of these gammaherpesvirus circRNAs can be found to play functional roles in virus infection.

**FIG 1:**
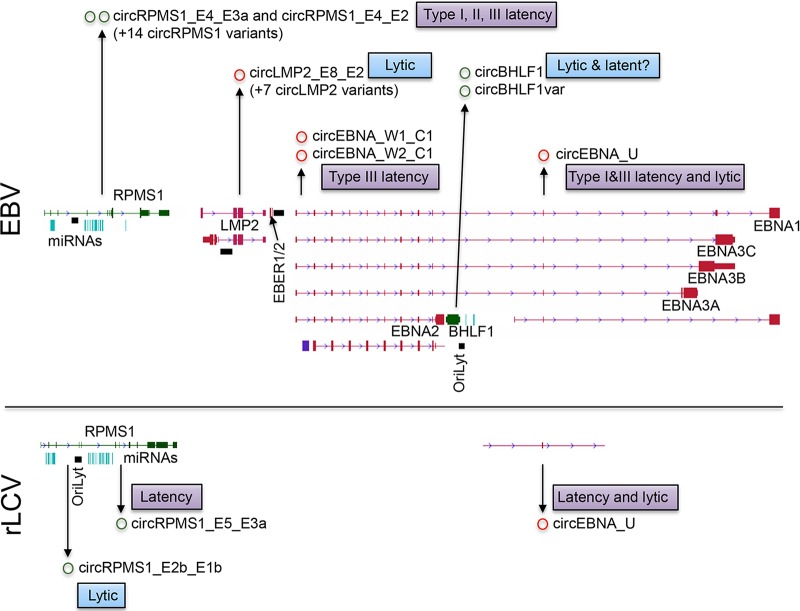
Key viral circRNAs identified for the lymphocryptoviruses EBV (top) and rLCV (bottom). Latency-coding genes (and corresponding circRNAs) are shown in red, and noncoding genes (and corresponding circRNAs) are shown in green. Aquamarine bars in RPMS1 loci represent viral microRNAs.

**FIG 2:**
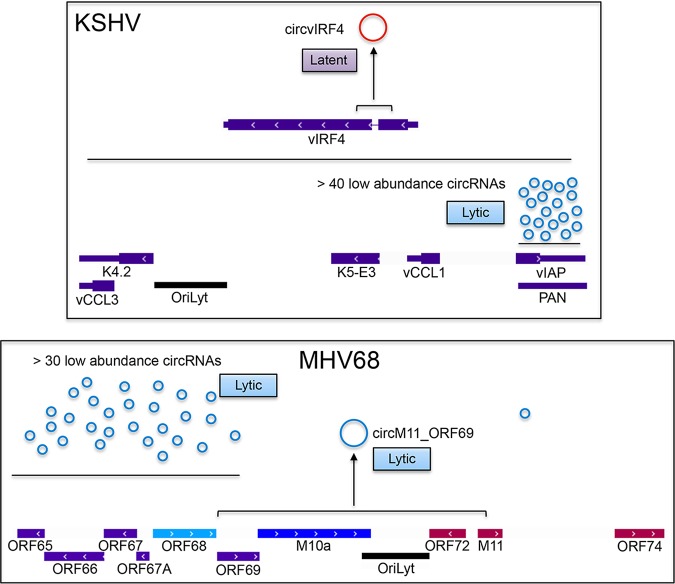
Key viral circRNAs identified for the rhadinoviruses KSHV (top) and MHV68 (bottom). Latency coding genes (and corresponding circRNAs) are shown in red. Immediate-early, early, and late lytic genes are represented by light, medium, and dark blue shades, with lytic circRNAs shown in medium blue.

While relatively diverse sets of low-abundance circRNA clusters were identified near the lytic origins of replication (OriLyts) in the rhadinoviruses KSHV and MHV68 (19–22), only one major abundantly expressed circRNA has been identified to date for each of these viruses (an abundant lytic circRNA derived from an MHV68 OriLyt and an abundant circRNA derived from the KSHV vIRF4 gene in latently infected cells [see Fig. 2]). In contrast, a much more extensive set of relatively abundant viral circRNAs have been identified in the lymphocryptovirus EBV (19), with a more limited study of an EBV relative, rhesus lymphocryptovirus (rLCV), also identifying multiple viral circRNAs (Fig. 1) (21). On the surface, the greater number of discovered EBV circRNAs is consistent with (i) the higher degree of viral linear splicing that is fundamental to the EBV latency gene structures, and/or (ii) the broader array of cell and latency types that have been investigated for EBV circRNAome studies relative to that of rLCV, KSHV, and MHV68. While the former explanation might be consistent with the “sporadic splicing mistake” contention, there are nevertheless arguments for functional significance. First, the evolutionary study by Ungerleider et al. (21) investigating the rLCV circRNAome (rLCV and EBV share nearly identical gene organization but only 65% nucleotide homology) in an *in vivo* rhesus macaque lymphoma model demonstrated conservation of two latency circRNAs: a circRNA expressed from the RPMS1 lncRNA locus and circEBNA_U, which is expressed from the EBNA latency locus (Fig. 1). Second, as discussed below, there is circumstantial evidence of homoplastic evolution of lytic circRNAs at OriLyt loci of all four viruses (21). Third, several viral circRNAs are derived from splice donors and/or acceptors that have not been found to be engaged in any linear splicing events, including KSHV circvIRF4, MHV68 circM11_ORF69, and EBV circW1_C1, and circW2_C1 circRNAs, which backsplice to a previously unknown spice acceptor located only 8 nucleotides downstream from the reported transcription initiation site, and EBV circBHLF1, which is the 5th most abundant circular RNA in the cell during reactivation. In the absence of any other need for these splice junctions, it is hard to imagine that purely wasteful metabolic events that backsplicing would represent would not be selected against, thereby raising the prospect of functional relevance. In line with the steady unraveling of cellular circRNA functions, it is likely that viruses, which are known for their rapid evolution and their efficient utilization of genomic resources, will similarly be found to have evolved with repertoires of circRNAs that serve various critical viral functions. At the same time, although more viral circRNAs may yet be discovered for KSHV and MHV68 in different cell models, it is likely that EBV’s evolutionary acquisition of more extensive, advanced, and complex splicing has concomitantly led to the coevolution of more extensive utilization of circRNAs. Whether rLCV lags behind in its acquisition of a similarly broad repertoire of circRNAs or whether further studies in different cell types, latency types, and during reactivation conditions will demonstrate conservation of most of the detected EBV circRNAs remains to be seen.

## MicroRNA SPONGES?

Although not pervasive among cellular circRNAs, probably the most acknowledged function of circRNAs is a microRNA sponge function (sequestration of microRNAs) (27). circRNAs that serve as microRNA sponges likely require a high per-cell molarity of microRNA binding sites, conferred through repetitive miRNA binding sequences and/or high-level expression of the respective circRNA (28). While the expression of some of the lytic EBV circRNAs, such as circBHLF1 and circBHLF1alt (Fig. 1), ranks highly compared to that of cellular circRNAs, Ungerleider et al. (19) utilized published photo-activatable ribonucleoside-enhanced cross-linking and immunoprecipitation (PAR-CLIP) data from 7 EBV-positive B-cell models and 2 KSHV-infected B-cell models and failed to identify miRNA interactions with EBV or KSHV circRNA sequences (19–22). While this does not exhaustively assess all settings from which EBV or KSHV circRNAs have been identified, it suggests that like cellular circRNAs, most viral circRNAs probably do not function as miRNA sponges.

## CODING FUNCTIONS?

The majority of cellular circRNAs have been shown to localize to the cytoplasm. Of the major EBV circRNAs shown in Fig. 1, circW1_C1, circW2_C1, circBHLF1, and circBHLF1alt are localized to the nucleus (19). Divergent PCR experiments using primers that amplify minimal regions surrounding the circLMP2_E8_E2, circRPMS1_E4_E3a, and circRPMS1_E4_E2 backsplice junctions showed mixed cytoplasmic and nuclear distributions (19). Interestingly, further structural analyses of circRPMS1_E4_E3a and circRPMS1_E4_E2 uncovered two isoforms for each, with one isoform containing an all exon-to-exon splicing configuration and the other retaining the intron between exons 3a and 3b. Consistent with previous studies showing that many intron-retained cellular circRNAs localize to the nucleus (26), the intron-retained isoforms of both circRPMS1_E4_E3a and circRPMS1_E4_E2 were found to be nuclear, whereas the intron-excised isoform localized to the cytoplasm (20). These findings explain the observed mixed distribution of circRPMS1_E4_E3a and circRPMS1_E4_E2 in the isoform agnostic backsplice PCR experiments mentioned above and broaden the spectrum of distinct functions for the different circRNA isoforms. Despite finding cytoplasmic localization for the intron-excised circRPMS1_E4_E3a and circRPMS1_E4_E2 isoforms, Toptan et al. (20) showed that these isoforms localize to the nonribosome fraction in ribosome fractionation experiments and are therefore likely not translated.

Like the circRPMS1_E4_E3a and circRPMS1_E4_E2 circRNAs, the KSHV circvIRF4 circRNA also shows both an intron-retained and an intron-excised isoform (Fig. 2) (20–22), and isoform agnostic PCR shows both nuclear and cytoplasmic distribution (20). At this point, it is not known whether the intron-retained isoform localizes to the nucleus while the intron-excised form localizes to the cytoplasm. However, like the observed lack of ribosomal localization of the cytoplasmic circRPMS1_E4_E3a and circRPMS1_E4_E2 isoforms, circvIRF4 did not localize to the ribosomal fractions (20). Together, these data support some parallels between the latently expressed KSHV circvIRF4 and EBV circRPMS1_E4_E3a and circRPMS1_E4_E2 circRNAs and, despite lacking any genetic similarity, raise the possibility of one or more cognate functions during latency.

The other viral circRNA that displays some level of cytoplasmic localization, circLMP2_E8_E2, is only detected during reactivation despite similar expression levels of the parental linear LMP2 transcript during latency (19). Interestingly, under latency conditions, two linear LMP2 isoforms are expressed, LMP2A and LMP2B, which differ in their utilization of alternative promoters/exon 1s (29), with the first exon of LMP2A encoding a cytoplasmic signaling domain and the first exon of LMP2B lacking any coding capacity. As such, circLMP2_E8_E2 contains all of the coding capacity of LMP2B (exons 2 through 8), raising the interesting possibility that circularization during reactivation represents an alternative strategy to generate an LMP2B protein isoform (19). Although a completely forward-spliced configuration of circLMP2 exons 2 through 8 was identified, an intron 4-retained isoform of circLMP2_E8_E2 has also been detected (N. A. Ungerleider, M. Concha, and E. K. Flemington, unpublished data), potentially explaining the mixed nuclear/cytoplasmic localization of circLMP2_E8_E2 (19). Whether the intron-retained isoform is nuclear and performs some nuclear function while the intron-excluded isoform is cytoplasmic and potentially expresses LMP2B protein remains to be determined.

## LYTIC CIRCLES AND OriLyts

The noncoding nature of viral circRNAs is particularly advantageous for the virus to elicit cell signaling during latency, where the virus exists under conditions of immunological stealth. Nevertheless, many gammaherpesvirus circRNAs are uniquely expressed during the lytic, replicative phase of their infection cascade (19–22). In this setting, hundreds of viral protein-coding genes are concurrently expressed without apparent deleterious immunological impact on this phase of infection. Without the severe restraints on viral protein expression that drives the utilization of numerous noncoding transcript types during latency, the utilization of noncoding viral circRNAs during the lytic cycle raises the possibility that circRNAs have unique effector functions that are more effectively achieved through circRNA-specific mechanisms.

While the comparison of EBV and rLCV circular RNA repertoires revealed conservation of the latency-associated EBV circRPMS1_E4_E3a and circEBNA_U circular RNAs (21), under lytic conditions, Ungerleider et al. (21) noted positional conservation of circRNA clusters at or near lytic origins of replication for all four viruses analyzed to date (Fig. 1 and 2). In EBV, the highly expressed circBHLF1 and its variant, circBHLF1var, are derived from a locus that is adjacent to one of the two OriLyts (19). Rennekamp et al. (30) previously showed that there are some incompletely characterized species of BHLF1 transcripts that interact with the adjacent OriLyt, and they showed that these interactions with OriLyt are essential for lytic DNA replication. While not tested, it is possible that the nuclear localized circBHLF1 and/or circBHLFvar mediate this interaction and promote replication. Assessment of the rLCV circular RNAome showed expression of linear BHLF1 transcripts but no evidence of circular BHLF1 transcripts (21). However, a novel viral lytic circRNA, circRPMS1_E2b_E1b, not found in EBV, was identified in rLCV that spanned the other lytic origin of replication (Fig. 1). In MHV68, a high-density cluster of low-abundance, lytic circular RNAs originating from a locus near an OriLyt and a highly expressed circRNA, circM11_ORF69, which spans this OriLyt (Fig. 2), was identified (21). In KSHV, a high-density cluster of poorly expressed lytic viral circRNAs derived from the PAN locus, located near a KSHV OriLyt, was identified (Fig. 2) (20–22). The finding of lytic circRNA clusters located at or near lytic origins of replication could be chance occurrences across all four viruses. On the other hand, it is possible that the act of lytic replication makes adjacent regions more susceptible to backsplicing, possibly through some unknown link in apparatuses involved in these two processes. Not mutually exclusive with this last possibility, the findings that BHLF1 transcripts engage in self-promoter regulation and the process of lytic DNA replication raises the possibility that these positionally similar viral circRNAs have active roles in facilitating OriLyt DNA replication.

## REGULATION OF VIRAL circRNA EXPRESSION

In humans, cellular circRNA formation has been shown to be driven to a large extent by the presence of flanking inverted Alu repeat elements that stabilize proximity of the splice donor and acceptor sequences (31). Such overtly identifiable inverted repeats are not present in the flanking introns of the gammaherpesvirus backsplice junctions identified to date, however. This does not preclude the possibility that unidentified smaller inverse complementary sequences are involved in promoting these backsplicing events, and Liang and Wilusz (32) have shown that as few as 36 bases of imperfect Alu complementarity can support cellular backsplicing. At the same time, splicing factors such as QKI and MBNL promote backsplicing through protein-protein interactions by binding to flanking intron sequences (33, 34), and it is possible that RNA binding/processing factors such as these play an important role in facilitating gammaherpesviral backsplicing.

While many gammaherpesvirus circRNAs show tissue-selective expression, this is primarily due to corresponding differences in the parental gene expression level, which varies according to infection stage and tissue type (Fig. 1 and 2). Nevertheless, some backsplice junctions display tissue selectivity that is not directly related to parental gene expression levels. Besides the constitutively expressed circRPMS1_E4_E3a and circRPMS1_E4_E2 EBV circRNAs that are expressed across all cell and latency (and reactivation) types, there are a number of additional RPMS1 circRNAs that are selectively expressed in reactivating Burkitt’s lymphoma cells but not latent stomach cancer settings that show comparable parental gene expression (19). circLMP2_E8_E2 is expressed specifically during reactivation despite similar levels of the parental LMP2 transcripts in other latency B-cell models (19). While the roots of EBV-specific lytic backsplicing are currently unknown, it is noteworthy that the EBV lytic transcriptome is highly complex, with alternative promoters and exons being utilized to express latency genes under lytic conditions (3, 35). The selective expression of some viral circRNAs during reactivation may therefore be related to unique *cis* elements in the primary lytic transcripts. Alternatively, backsplicing at these loci may be regulated by tissue-specific and/or virus-encoded splicing factors, such as the EBV lytic splicing/transport factor BMLF1. Interestingly, a converse scenario is observed with the KSHV circvIRF4 circular RNA (20–22). The parental linear vIRF4 transcript is expressed at low levels during latency, and its expression is substantially increased during reactivation. Nevertheless, circvIRF4 displays latency expression, and its expression decreases following reactivation (20–22). The lack of circvIRF4 induction under conditions where the apparent parental transcript is highly induced could be due to distinct *cis* elements present uniquely in the primary latency and lytic vIRF4 transcripts or could be the consequence of other mechanisms, such as the presence of backsplicing suppressors that limit circularization during reactivation.

## FINAL THOUGHTS

To minimize immune recognition of the infected cell during long-term persistence, gammaherpesviruses have evolved with latency gene expression programs that utilize noncoding RNAs to affect the host environment. Understanding these latency phases of gammaherpesvirus infection is important, because it speaks to the mechanisms of long-term persistence and the mechanisms through which these viruses contribute to cancer. While latency-associated long and short noncoding RNAs have been important topics of investigation, we have just now learned that these viruses also encode the circular RNA class of noncoding RNAs. This adds a new layer to the arsenal of noncoding RNAs that are potentially utilized by these viruses to influence the host cell environment and opens up a new field of investigation that is likely to be highly germane to virus biology and pathogenesis. At the time of this writing, we have been unable to find reports of circRNAs expressed from the alpha- or betaherpesvirus subfamilies (although the herpes simplex virus LAT RNA has been shown to generate a stable intronic lariat structure, a covalently closed RNA distinct from circRNAs in the mechanism of generation and the 2′ to 5′ versus 3′to 5′ covalent end joining [36]). It is possible that there are unique aspects of the gammaherpesvirus infection cycle that specifically drive gammaherpesvirus’ selective utilization of circRNAs. On the other hand, given the recency of gammaherpesvirus circRNA discoveries, the discovery of circRNAs encoded by these other subfamilies may simply be a matter of time.

Critical to new investigations of viral circRNAs will be studies into their functions and mechanisms of action. It will also be relevant to gain an appreciation for possibly unique mechanisms through which viral circRNAs are generated and the relationships and interactions between cognate linear transcript splicing (and possibly microRNA processing) and circRNA splicing. Understanding the mechanisms driving the formation of viral circular RNAs will not only inform us on fundamental molecular biology of circRNA formation but also will contribute to our understanding of their function, since protein factors involved in facilitating backsplicing load onto the circRNA and are critical for their localization and downstream functions. It will be fascinating to monitor investigations into these newly appreciated viral noncoding RNAs in the coming years, as we gain insight into fundamental virus biology, the mechanisms driving virus persistence in the host, and the mechanisms through which gammaherpesviruses promote cancer.

Lastly, because of their stability, there is a fair amount of interest in circRNAs as potential liquid/blood biomarkers of disease (18, 37). Some viral circRNAs identified to date, most notably, circRPMS1_E4_E3a and circRPMS1_E4_E2, are expressed in latency and have been detected in virus-associated patient tumor samples (19–22). This places them as potential biomarkers for virus-associated cancers. Further, there is even the possibility that tissue-, latency type-, and reactivation-specific viral circRNA expression allows for the determination of disease-associated infection types through blood-based detection of viral circRNAs. In addition to the importance of investigations into the roles of viral circRNAs in virus biology and cancer signaling, it will also be important to determine whether viral circRNAs represent viable therapeutic targets and whether they provide diagnostic utility.
